# Editorial: Advancing understanding of neonatal bacterial infections

**DOI:** 10.3389/fcimb.2025.1746698

**Published:** 2025-12-11

**Authors:** Susana Chavez-Bueno, Shelley M. Lawrence

**Affiliations:** 1 Division of Pediatric Infectious Diseases, Department of Pediatrics, Children’s Mercy, Kansas City, MO, United States; 2 University of Missouri Kansas City (UMKC) School of Medicine, Kansas City, MO, United States; 3 Department of Pediatrics, Division of Neonatology, Spencer Fox Eccles School of Medicine, University of Utah, Salt Lake City, UT, United States; 4 Primary Children’s Hospital, Intermountain Healthcare, Salt Lake City, UT, United States

**Keywords:** neonatal sepsis, antimicrobial resistance (AMR), antimicrobial stewardship (AMS), neonates, microbiome & dysbiosis, heparin binding protein (HBP), animal models, bacterial infections

Sepsis is broadly defined as a life-threatening host response to infection leading to organ dysfunction (GBD 2021 Global Sepsis Collaborators, 2025). However, newborns often exhibit organ dysfunction that inversely correlates with their gestational age at birth, underscoring the critical role of their developing immune system in combating overwhelming infection. Presently, there is no consensus definition for neonatal sepsis, and the International Consensus Criteria for Pediatric Sepsis and Septic Shock, published in 2024, does not pertain to preterm infants less than 37 weeks of gestation (Schlapbach et al., 2024).

This Research Topic aims to enhance the comprehension of the molecular pathogenesis of bacterial infections in newborns and to explore the diverse immune responses elicited by these pathogens. Increased understanding of the individual host-pathogen interactions that determine clinical outcomes will contribute to reducing the incidence and severity of bacterial infections in this vulnerable population. Moreover, continued monitoring of global epidemiological trends of neonatal bacterial infections is key to adapting effective approaches to prevent, diagnose, and treat neonatal sepsis in specific settings and also at a large scale.

Globally, it is estimated that nearly 5.4 million neonates, or infants aged ≤28 days, will develop sepsis, and approximately 1 million will succumb to sepsis-related complications (GBD 2021 Global Sepsis Collaborators, 2025). Improvements in neonatal care have led to reductions in neonatal sepsis-related mortality from 53.9% of all deaths in 1990 to 44.4% in 2021. This was the most substantial decline among any patient group (GBD 2021 Global Sepsis Collaborators, 2025). Regional differences in the burden of neonatal sepsis remain. As reported by Mu et al., morbidity and mortality remain high in Africa and Southeast Asia, and are increasing in the Eastern Mediterranean and Western Pacific regions. This trend is alarming, as antimicrobial resistance (AMR) is escalating, with nearly one-third of gram-negative bacteria exhibiting carbapenem resistance in Africa (Sisay et al., 2024). In Northeast Ethiopia, Shimeles et al. showed an increased risk of neonatal sepsis in women with vaginal colonization by pathogenic bacteria, including *Escherichia coli*, *Candida* spp., and *Klebsiella* spp., with multidrug resistance observed in 37.3% of cases. Additionally, up to 97% of Gram-negative bacteria in this region are nonsusceptible to ampicillin, and 70% to gentamicin, although these antibiotics remain primary choices for empiric coverage (Harrison et al., 2024).

The use of inconsistent methodologies in the investigation of neonatal sepsis, coupled with insufficient research funding, hampers our capacity to accurately define its incidence and to ensure that potential treatments and clinical interventions are rigorously investigated. Consequently, the medical management of neonatal sepsis, septic shock, and multisystem organ dysfunction is predominantly guided by protocols established for critically ill pediatric and adult patients. Despite these limitations, antimicrobial stewardship initiatives have led to hospital-specific modifications to the recommended duration of antibiotic therapy for neonatal sepsis and pneumonia, as established by the American Academy of Pediatrics and the Committee on Infectious Diseases, as highlighted by Lawrence et al. Alarmingly, these modified treatment regimens are often implemented without parental consent, consideration of the infant’s gestational age, regard for the causative bacteria, or evidence of long-term safety.

Improved testing methods are necessary for more precise identification of neonatal sepsis. Increasing diagnostic accuracy is key to better determine which newborns will benefit the most from receiving empiric antibiotic therapy, particularly those born preterm. Numerous biomarkers have been proposed as ancillary tools for the diagnosis of sepsis, but no individual test has been deemed sufficiently accurate to be recommended for routine clinical application. Chen et al. present data on the role of heparin-binding protein (HBP) as a promising biomarker for early diagnosis and severity assessment of neonatal sepsis. HBP is significantly elevated in neonates with severe sepsis. The authors highlight the existing evidence demonstrating that HBP outperformed traditional markers like procalcitonin and high-sensitive C-reactive protein in the diagnostic accuracy of neonatal sepsis. Moreover, their manuscript presents evidence that in older populations, HBP aids in differentiation between patients with bacterial vs. non-bacterial infections, potentially helping to guide more rational antibiotic use. Studies show HBP’s sensitivity and specificity are high at various cut-off values for the diagnosis of sepsis in adults, making it a promising biomarker that necessitates further evaluation in neonatal populations for this application.

Another article in this Research Topic presents data on the characterization of the host response to infections in neonates, which can aid in the diagnosis of sepsis. Gharaibeh et al. analyzed the differential expression of 100 genes in neonates with sepsis to establish a transcriptomic mortality signature. Gene profiling distinguished the infants into three well-defined groups, with endotype “A” exhibiting the highest mortality rate. A dysregulated hyperinflammatory response accompanied by emergency granulopoiesis was characteristic of these infants, potentially informing the necessity for targeted sepsis therapies.

In addition to the relevant role of innate immune responses in the pathogenesis of neonatal bacterial infections, the interaction of commensal and pathogenic bacteria within the host also determines important clinical outcomes. In this compilation, human trials were conducted to assess potential associations between the placental microbiome and neonatal pneumonia. Zhang et al. demonstrated that placentas with reduced levels of *Lactobacillus* were more likely to exhibit increased abundance of *Ureaplasma* and *Staphylococcus*, a profile positively correlated with neonatal pneumonia in late-preterm infants.

While human clinical trials can provide considerable insights into disease pathophysiology, they are often complicated by the heterogeneity of disease presentation. Consequently, the development of validated animal models for neonatal sepsis remains essential. Sellers-Porter et al. characterized their murine model of neonatal sepsis using a stock slurry from an infant who died of necrotizing enterocolitis. These investigators demonstrated reproducibility and validity of their model, showing that inflammation, sepsis-induced mortality, and intestinal injury were dependent upon the concentration of live bacteria administered and attenuated by antibiotic administration. This murine model may inform pathophysiologic differences between neonatal and adult mice, thereby facilitating testing of potential interventions.

Alternatively, the investigation of adjunctive therapeutics concurrent with antibiotics was tested in a neonatal murine model of *E. coli* sepsis. In this investigation, Speer et al. detailed renal tissue damage and inflammation, showing that acute kidney injury could be reduced by coadministration of pentoxifylline with targeted antibiotics. This combination therapy also decreased plasma levels of interleukin-6, tumor necrosis factor-alpha, and neutrophil gelatinase-associated lipocalin (NGAL), among others. Additional studies are needed to determine the optimal therapeutic approach, which will involve modulating host responses to infection in conjunction with the use of targeted, effective antimicrobial agents.

In summary, the field of neonatal sepsis is continually evolving, with the goal of developing improved, personalized diagnostic approaches and more effective treatment strategies. To ensure equitable outcomes, these interventions must be tailored to diverse populations and settings around the world.
